# Unsupervised adaptation of an ECoG based brain–computer interface using neural correlates of task performance

**DOI:** 10.1038/s41598-022-25049-w

**Published:** 2022-12-09

**Authors:** Vincent Rouanne, Thomas Costecalde, Alim Louis Benabid, Tetiana Aksenova

**Affiliations:** 1 Univ. Grenoble Alpes, CEA, LETI, Clinatec, 38000 Grenoble, France; 2 CHU Grenoble Alpes, Grenoble, France

**Keywords:** Neuroscience, Biomedical engineering

## Abstract

Brain–computer interfaces (BCIs) translate brain signals into commands to external effectors, and mainly target severely disabled users. The usability of BCIs may be improved by reducing their major constraints, such as the necessity for special training sessions to initially calibrate and later keep up to date the neural signal decoders. In this study, we show that it is possible to train and update BCI decoders during free use of motor BCIs. In addition to the neural signal decoder controlling effectors (control decoder), one more classifier is proposed to detect neural correlates of BCI motor task performances (MTP). MTP decoders reveal whether the actions performed by BCI effectors matched the user’s intentions. The combined outputs of MTP and control decoders allow forming training datasets to update the control decoder online and in real time during free use of BCIs. The usability of the proposed auto-adaptive BCI (aaBCI) is demonstrated for two principle BCIs paradigms: with discrete outputs (4 classes BCI, virtual 4-limb exoskeleton), and with continuous outputs (cursor 2D control). The proof of concept was performed in an online simulation study using an ECoG dataset collected from a tetraplegic during a BCI clinical trial. The control decoder reached a multiclass area under the ROC curve of 0.7404 using aaBCI, compared to a chance level of 0.5173 and to 0.8187 for supervised training for the multiclass BCI, and a cosine similarity of 0.1211 using aaBCI, compared to a chance level of 0.0036 and to 0.2002 for supervised training for the continuous BCI.

## Introduction

BCIs have several well-known drawbacks that prevent them from being used in day-to-day scenarios. Many of these drawbacks are associated with the necessity to train BCI decoders. Decoders in BCIs are mostly trained using supervised learning^1^. Supervised learning requires having access to labels in addition to neural data. In the case of supervised learning for BCIs, it is required to know the user’s intentions during the decoder-training phase in order to derive labels. In order to know their intentions, BCI users are constrained to perform specific actions when training their BCI decoders. This decoder training scheme is an issue for several reasons. Firstly, the user’s brain signals may differ between actions they were directed to perform and actions they freely decided to perform, leading to differences between the dataset the decoder is trained on and the one it is used on. Secondly, having access to the user’s intentions requires a training environment in which it is possible to direct them to perform well-defined actions. Controlled environments are especially important for complex motor tasks, including the control of multi-dimensional continuous output. In the current article, we refer as ‘complex BCI’, BCI systems with continuous control of at least two degrees of freedom (e.g. 2D or 3D hand movements). Thirdly, the user cannot freely choose actions to be performed during the training or updating periods. Thus, the BCI cannot be used for its intended purpose during these periods. This time loss increases with the complexity of the output of the BCI^2,3^. Finally, the performances of BCI decoders can fluctuate with time for several reasons, such as variations in the user’s psychological state, plasticity or other concept drifts. The BCI decoding efficiency degradation may in turn require updating the decoders or training new decoders from scratch more or less frequently^4,5^.

Several strategies were developed to try to remove the need for training sessions, or at least reduce the decoder training time as much as possible. Transfer learning can reduce or remove the need for training sessions for new users by directly using or updating decoders trained for previous users. Transfer learning can also be used to train decoders on new tasks using data from previous tasks^6^. However, transfer learning does not prevent decrease of performances due to concept drifts and transfer learning between users has not been achieved yet for complex motor decoding tasks. Another strategy is to train control decoders using unsupervised learning instead of supervised learning, as it does not require labeled data to train or update a decoder^7^. However, unsupervised learning is more suited for updating decoders that were already trained rather than training new decoders^1^. Additionally, fully unsupervised learning can be used to train simple classification tasks^8^, but has not been used on complex BCIs with continuous effector control through regression.

**Figure 1:**
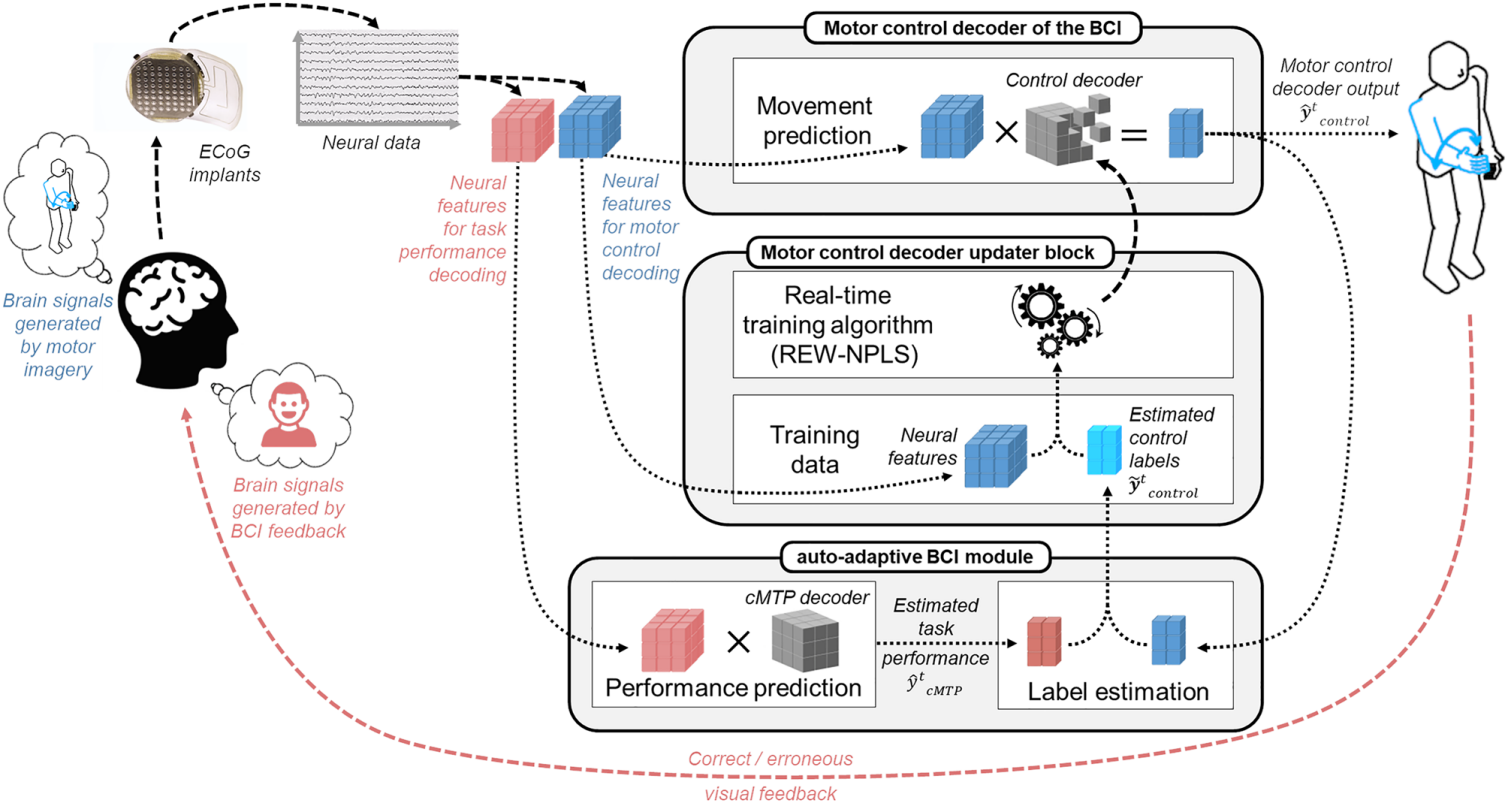
Diagram of the aaBCI framework. In addition to the control decoder block and the updater block common to most BCIs, the aaBCI features an auto-adaptive BCI module. The aaBCI module is responsible for the detection of neural correlates of continuous in time motor task performance (cMTP), as well as the estimation of labels for the control decoder (${{\widetilde{\mathbf{y}}}^{\mathrm{t}}}_{\mathrm{control}}$) based on the output of the cMTP decoder (${{\widehat{\mathrm{y}}}^{\mathrm{t}}}_{\mathrm{cMTP}}$) and the output of the control decoder (${{\widehat{\mathbf{y}}}^{\mathrm{t}}}_{\mathrm{control}}$).

The approach proposed in this study is to infer labels during self-directed use of the BCI by the user. We detect neural correlates to evaluate how accurately actions performed by the BCI’s effectors matched the user’s intentions. Such neural correlates are referred as neural correlates of BCI control task performance. This knowledge is then combined with the corresponding outputs of the control decoder in order to create estimated labels. These estimated labels can be used to train from scratch or update the decoder used to control the BCI (control decoder). We refer to this type of BCI as “auto-adaptive BCI” (aaBCI) since the BCI’s decoder can be trained or updated adaptively while its effectors are being freely controlled. The auto-adaptive BCI framework is depicted in Fig. 1.

The idea of using neural correlates of BCI control task performances for adaptation of control decoder was already discussed in the literature^9^. However, to our knowledge, auto-adaptive BCIs are currently limited to communication BCIs such as spellers based on classification tasks. Adaptation of complex motor BCIs using neural correlates of task performance has not been achieved yet. Auto-adaptive BCIs using task performance levels were performed in simulation studies^7,10–13^ where the task performance signal was not evaluated from neural data but simulated. These simulation studies were limited to BCIs used for binary classification, with the exception of a study by Gürel and Mehring^7^ where a task performance signal is proposed to be used to perform adaptation of a decoder with a multi-dimensional continuous output. However, this study was performed in fully simulated conditions, i.e. their simulated continuous effector was controlled by a stochastic optimal controller simulating a user and not by the decoding of neural patterns of a real subject.

In addition to simulation studies, auto-adaptive BCIs were also implemented using a neural correlate of BCI control task performance called the Error Related Potential (ErrP). The ErrP is a waveform that is time-locked to an event (event-locked) regarded as erroneous. Both offline^12^ and online^8^ studies were performed, but were limited to the update of decoders used for classification problems.

Advanced motor BCIs require relatively high control and feedback rates^14^ including both discrete and continuous outputs^2^. Adaptive strategies for motor BCIs with continuous outputs have different constraints than adaptive strategies for BCIs with discrete outputs. For BCIs with discrete outputs, a signal reflecting the task performance event-locked to specific events, such as changes in the classification output, may be sufficient. Inversely, neural correlates of task performance that are event-locked are ill-suited to continuous BCIs. Indeed, BCIs with continuous outputs can have variations in their output at each (short) time step, which are unlikely to all elicit event-locked neural correlates. Detection of error correlates during continuous tasks was studied by Omedes et al.^15^ and Lopes-Dias et al.^16^. However, in the first study decoders trained to detect gradually unfolding errors during a 2D continuous task failed to perform better than chance level. In the second study, participants controlled a robotic arm in a 2D plane, and errors where artificially introduced as loss of control over the robot along with a displacement of the robot in the direction orthogonal to the plane of control. Although the effector was controlled in a two-dimensional plane, the erroneous events were discrete events and are thus comparable to the ones that would occur in non-complex BCIs. For complex motor BCIs, we suggest that continuous in time motor task performances (cMTP) evaluation should be used instead of performances evaluation obtained only at the time of certain events (event-locked motor task performance, eMTP), such as task outcome or sub-task outcomes (e.g. ErrPs). Access to cMTP is more powerful than access to eMTP as it provides an evaluation of performances for each time point, where eMTP would need to extrapolate performances around measure points. For complex tasks, this extrapolation would not hold (e.g. attaining a target in a reaching task does not ensure every step of the reaching trajectory was accurate). For this reason, we argue that in the context of auto-adaptive BCIs cMTP should be preferred over eMTP when available, and especially for complex motor BCIs.

The optimal brain region for the detection of cMTP neural correlates is up to discussion. ErrP is the most studied neural correlate of task performance. Although no studies investigated it yet, the brain structures responsible for ErrPs might also generate cMTP neural correlates. The different components of ErrPs are thought to originate from the medial and middle frontal gyrus, the gyrus postcentralis and the anterior cingulate cortex^17^. However, this recording area is not compatible with current state-of-the-art motor BCIs. For now, BCIs that provide accurate control over multiple continuous degrees of freedom use neural correlates of motor imagery^2,18^ and are limited to recordings from the sensorimotor cortex. cMTP correlates should be derived from neuronal activity recordings of the sensorimotor cortex in order to be useful for current state-of-the-art BCIs. Evidence of eMTP correlates in the sensorimotor cortex were produced at several occasions^19–23^. Interestingly, these eMTP correlates were reported largely in the time–frequency domain rather than the temporal domain like ErrPs. However, to date no study reported the existence of cMTP correlates in the sensorimotor cortex. Detection of cMTP neural correlates in the sensorimotor cortex was the first objective of the study.

Regardless of which cerebral structure they are obtained from, the aaBCI framework estimates cMTP from neural recording. Estimated cMTP is then used to create labeled training samples to calibrate or update the BCI control decoder. Samples that are estimated as correct or erroneous with high certainty are combined in real-time with their respective output from the control decoder to create estimated labels. The aaBCI uses a training algorithm that can run online and in real-time in order to use estimated labels for online adaptation of control decoders during self-directed use of the BCI. The second objective of the study was to perform proof of concepts of the auto-adaptive BCI, and to test its performances during simulated online use.

**Figure 2:**
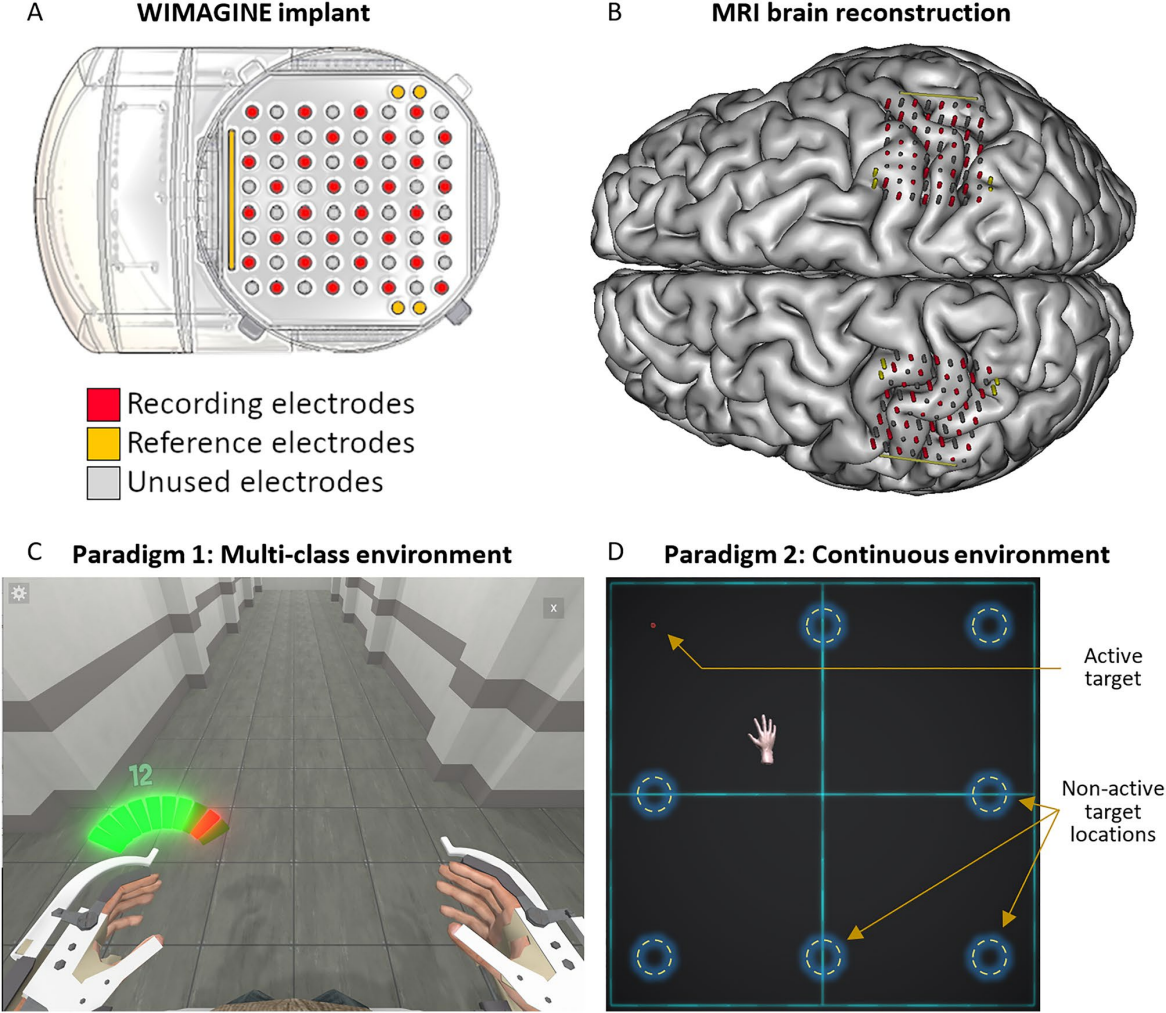
(**A**) Schematic view of a WIMAGINE implant. (**B**) Position of the electrodes of each WIMAGINE implant over the right and left sensorimotor cortex on a reconstruction of the subject’s brain from MRI. (**C**,**D**) Virtual environments used for each series of experiment. (**C**) The multi-class discrete BCI experiment (paradigm 1) was done with a virtual avatar in an exoskeleton seen from a first-person point of view. The subject used motor imagery to activate and maintain four mutually exclusive movement states: rotation of the left or right wrist or movement of the left or right hand. (**D**) The bi-dimensional continuous BCI experiment (paradigm 2) was done with a two-dimensional center-out task on a computer screen. The subject used motor imagery to control continuously in two dimensions the direction and velocity of the hand-shaped cursor.

To perform this study, two datasets containing ECoG recordings from the left and right sensorimotor cortex of a tetraplegic subject enrolled in the clinical trial “BCI and tetraplegia”^2^ were acquired. Each dataset contained data from one of two motor BCI paradigms: a BCI with multiple discrete outputs (1st paradigm) and a BCI with multiple continuous outputs (2nd paradigm). The BCI with multiple discrete outputs was a 4-class BCI using a virtual 4-limb exoskeleton displayed on a computer screen as an effector (Fig. 2C). The subject had to use motor imagery to activate and maintain four mutually exclusive movement states: movement of the right or left hand or rotation of the right or left wrist. The BCI with multiple continuous outputs controlled a 2D hand-shaped cursor on a computer screen (Fig. 2D). The subject used motor imagery to control the velocity and the direction of the cursor in a center-out task. The control commands were sent to effectors every 100 ms. The control decoders used to record these datasets were trained classically during dedicated training sessions. Ten sessions of approximate duration of 20 min were performed over 86 days (31.10.2019–24.01.2020) with paradigm 1. Nineteen sessions of average duration of 16 min were performed over 47 days (21.08.2017–06.10.2017) with paradigm 2.

Neural feature extraction was performed on the ECoG signals by a time–frequency decomposition of one-second epochs, in 15 frequency bands centered from 10 to 150 Hz and from 64 electrodes over the left and right sensorimotor cortex. cMTP decoders were trained using these neural features and the N-way Partial Least Squares algorithm^24^. For both BCI paradigms, the output of the cMTP decoder was combined with the output of the control decoder to create training data which was in turn used to train the control decoders of the BCI in simulated real-time using the recursive exponentially weighted NPLS (REW-NPLS) incremental adaptive algorithm^25^. For both paradigms, to evaluate the efficiency of the proposed approach, we compared the performance of control decoders trained with the aaBCI framework (using labels estimated from the output of the cMTP decoder), to decoders trained in a supervised manner (using true labels) and with decoders trained using the auto-adaptive BCI but with the outputs of the cMTP decoders randomly shuffled (chance level) in a pseudo-online simulation.

## Results

### Detection of cMTP neural correlates in the sensorimotor cortex

The capacity of the cMTP decoder to estimate accurately if the BCI control command was correct or erroneous was evaluated for each motor BCI paradigm. The mean and standard deviation of the area under the curve (AUC) of the receiver operating characteristic (ROC) curve of the cMTP decoder over a five-fold cross validation were 0.5677 ± 0.0427 for paradigm 1 (4-class BCI) and 0.6479 ± 0.0202 for paradigm 2 (bi-dimensional continuous BCI).

### Estimation of labels for control decoder update using the cMTP output

**Figure 3:**
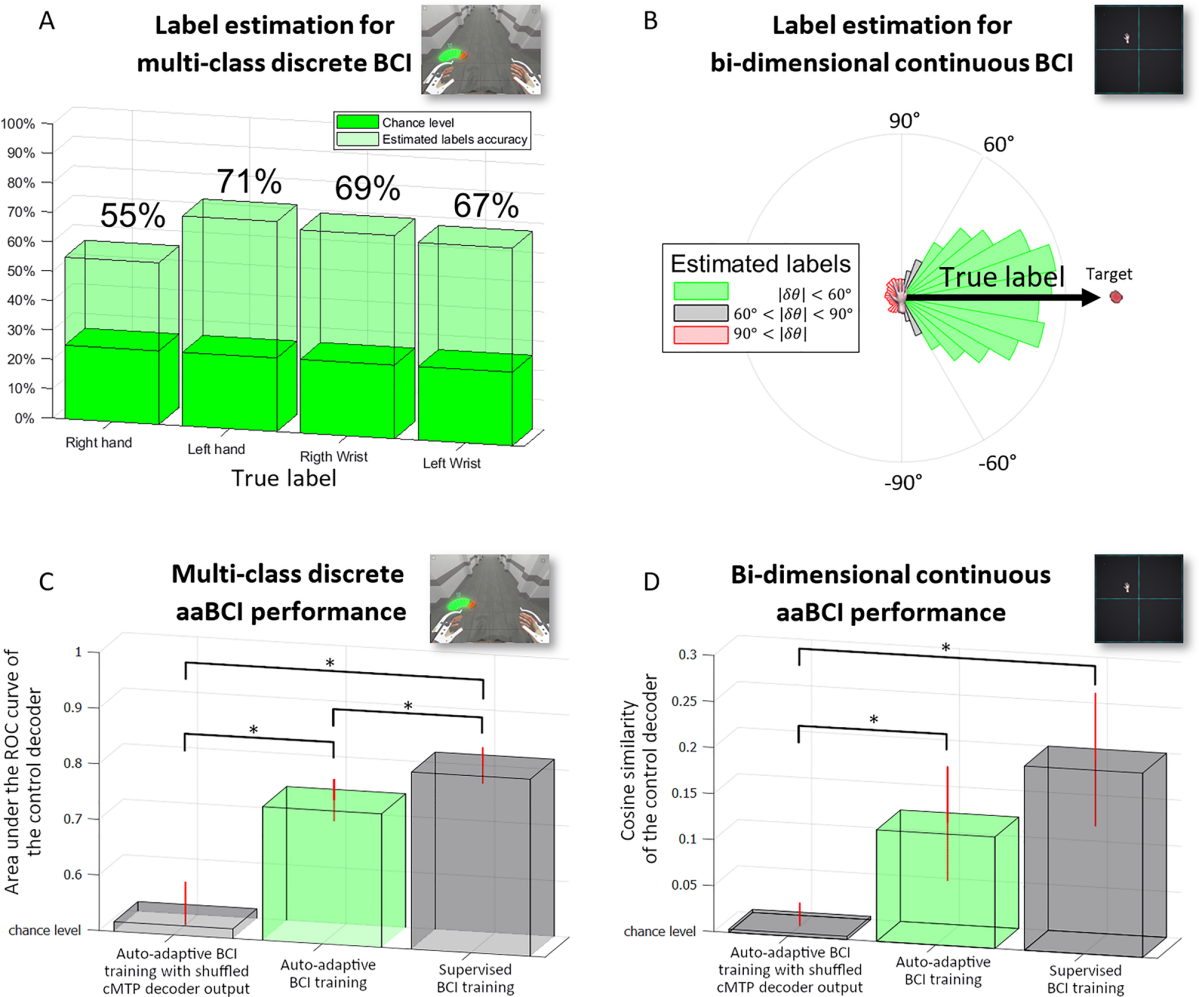
(**A**,**B**) Accuracy of the estimated labels in the datasets used for the training of the aaBCI control decoders. (**A**) Percentage of labels correctly estimated for each motor class for the discrete multi-class BCI. (**B**) Polar histogram of the angle between estimated control labels (using the aaBCI, ${{\widetilde{\mathbf{y}}}^{\mathrm{t}}}_{\mathrm{control}}$) and the actual labels (the $\mathrm{x}$ and $\mathrm{y}$ Cartesian distance from the cursor to the target, ${{\mathbf{y}}^{\mathrm{t}}}_{\mathrm{control}}$). When labeling data for the cMTP decoder, epochs with angles between the movement performed (${{\widehat{\mathbf{y}}}^{\mathrm{t}}}_{\mathrm{control}}$) and the actual labels (${{\mathbf{y}}^{\mathrm{t}}}_{\mathrm{control}}$) that were higher than 90° were labeled as erroneous, epochs with angles lower than 60° were labeled as correct and epochs with anglens between 60° and 90° were discarded. (**C**,**D**) Comparison of the performance of the control decoder depending on its training method. Error bars represent standard deviations. Stars denote statistically significant differences (two-sided Wilcoxon–Mann–Whitney tests, p < 0.005). (**C**) In the multiclass discrete BCI paradigm, performances were evaluated using the multi-class generalization of the area under the curve (AUC) of the receiver operating characteristic curve. (**D**) In the bi-dimensional BCI paradigm, performances were evaluated using the cosine similarity between decoded and optimal cursor trajectories.

Only the samples estimated as correct or erroneous with high certainty were combined with outputs from the control decoder to estimate labels for the control decoder update. This strategy effectively boosts the performance of the cMTP decoder on the epochs included in the dataset for the update of the control decoder. In the simulation of online use of the aaBCI, the data added to the training pool was decoded correct or incorrect by the cMTP decoder with an accuracy of 70.5% ± 8.1% for paradigm 1 (the multi-class BCI). Similarly, the data added to the training pool using the bi-dimensional continuous BCI (paradigm 2) was labeled correct or incorrect by the cMTP decoder with an accuracy of 71.5% ± 7.4%. The estimated control decoder labels had a global accuracy of 64.5% in paradigm 1 (Fig. 3A). In paradigm 2, the estimated control decoder labels were considered correct when the angle between the estimated label and the true label was less than 60°. The estimated control decoder labels had an accuracy of 63.3% (Fig. 3B).

### Simulation of online use of the aaBCI

The capabilities for auto-adaptive training of control decoders in the aaBCI framework was evaluated in an online simulation. The decoding capabilities of the control decoder of the multi-class BCI (paradigm 1) were assessed using a generalized version of the AUC of the ROC for non-binary classification^26^. The decoding capabilities of the control decoder of the continuous bi-dimensional BCI (paradigm 2) were assessed using the cosine similarity between the prediction (control decoder output) and the optimal prediction. The optimal prediction at each time moment is a Cartesian vector between the current cursor position and the target position^3^.

For paradigm 1, the mean and standard deviation of the AUC of the ROC were 0.7404 ± 0.0379 (Fig. 3C). For paradigm 2, the mean and standard deviation of the cosine similarity were 0.1211 ± 0.0623 (Fig. 3D). As a comparison, with an aaBCI update perfomed with a shuffled cMTP decoder output these results were 0.5173 ± 0.0603 for the multi-class BCI and 0.0036 ± 0.0217 for the continuous BCI, wheras with an update performed with fully supervised learning using true labels these results were 0.8187 ± 0.0324 for paradigm 1, and 0.2002 ± 0.0725 for paradigm 2.

There was a significant effect of the training method (supervised vs auto-adaptive vs random cMTP auto-adaptive) on the decoding capabilities of the control decoder for both paradigms (Friedman test, p = 0.0025). As expected, control decoders trained in a supervised manner performed significantly better than chance levels (two-sided Wilcoxon–Mann–Whitney tests, multiclass paradigm p = 0.0022, continuous paradigm p = 0.0022). More interestingly, control decoders trained with the aaBCI performed significantly better than chance levels (two-sided Wilcoxon–Mann–Whitney tests, multiclass paradigm p = 0.0022, continuous paradigm p = 0.0022). Control decoders trained with the aaBCI performed worse than the control decoders trained in a supervised manner. This difference was significant for the multiclass BCI paradigm, but not for the continous one (two-sided Wilcoxon-Mann–Whitney tests, p = 0.0043 for paradigm 1, p = 0.0649 for paradigm 2).

## Discussion

In this study, we reported the successful detection of cMTP neural correlates in ECoG data recorded from the sensorimotor cortex. There is a rational behind the presence of motor task performance neural correlates in the sensorimotor cortex. Modern computational models of the basal ganglia treat it as a part of a reinforcement-learning network for motor actions: mismatches between predicted movements and the reality are encoded in the spiking rate of midbrain dopaminergic neurons^27^. Furthermore, the basal ganglia projects into the primary motor cortex through the thalamus. The role of the basal ganglia in motor adaptation and its projections into the motor cortex could justify the presence of MTP neural correlates in the motor cortex.

Detection of cMTP neural correlates in ECoG data was demonstrated during control of two different effectors by a BCI user. The ability to obtain from the sensorimotor cortex a continuous in time signal correlated to the real-time performance of the BCI’s effector control is a novelty. As discussed previously, fast-paced continuous in time BCI task performance evaluation is more valuable for the purpose of the auto-adaptation of control decoders than the outcome-time-locked responses classically studied in the literature (such as the ErrP). This is especially true for continuous trajectory control tasks, where the outcome of a task does not provide a measure of performance of the control at each point of the trajectory whereas cMTP neural correlates do.

The proof of concept performed in this study demonstrated that the auto-adaptive BCI framework could be used for the training of control decoders of both discrete (classification based) and continuous control BCIs. As expected, the performances of aaBCI-trained control decoders were lower than when trained in a supervised manner. Indeed, the estimated labels used in the aaBCI framework are bound to have some uncertainty due to the cMTP decoder not having perfect accuracy. In contrast, the labels used in fully supervised training are accurate if we assume that the user properly performs the tasks demanded. This difference in label accuracy is at least partly responsible for the difference in performances between supervised and aaBCI decoder training. Additionally, the aaBCI training scheme discards samples that are not considered correct or erroneous with enough certainty leading to a difference in the number of samples used for the training of the supervised control decoder and the aaBCI one. The size difference between the training datasets may account for a part of the difference in performances between the two training methods. However, we hypothesize that in the long-term the performance gap between aaBCI and supervised-based training may be smaller. During self-directed use, the aaBCI would still be able to label data and use it to update its control decoder, whereas a control decoder trained supervisedly would remain fixed. The size of the training dataset of an aaBCI-trained control decoder will eventually overpass the size of supervised training datasets. Furthermore, control decoders trained using the aaBCI could suffer less from concept drifts since they are updated continuously, as long as the cMTP decoder is valid.

The name auto-adaptive BCI reflect the ability of the BCI system to create labeled data automatically for its control decoder during its free use, and distinguish the proposed strategy from classical fully unsupervised approaches. However, the aaBCI framework proposed in this study is not a fully unsupervised system since labeled data are needed to train the cMTP decoder. As the cMTP decoder is trained in a supervised manner, a dedicated training session is still required. Nevertheless, even if the cMTP decoder has to be updated or retrained occasionally, updating the binary cMTP decoder should be easier and faster than updating or retraining complex control decoders that control larger number of degrees of freedom. Additionally, the cross-validation scheme used in this study suggest that the cMTP correlates detected in the sensorimotor cortex are at least partly robust over time. The cross-validations folds were composed of distinct recording sessions, therefore the cMTP decoder was not always trained and tested on temporally close datasets. This property is valuable for the long-term adaptive capabilities of the aaBCI, as it reduces the need to update regularly the aaBCI’s cMTP decoder. Interestingly, the robustness in time of the cMTP neural correlates detected here in the sensorimotor cortex is consistent with the robustness of the classically studied error-related neural correlate, the Error-Related Potential^28,29^. Further studies should thoroughly quantify the robustness of cMTP decoders over time.

Several aspects of the aaBCI proposed here can be improved upon in future studies. The feature extraction should be refined as more studies investigate neural correlates of cMTPs. The labelling process could be elaborated further, especially for continuous BCI paradigms where the method used here only made use of samples estimated as correct. Moreover, epochs are considered as correct for deviations up to 60 degrees away from the ideal trajectory. Other labeling strategies should be tested.

To approach medical use, more complex BCI paradigms with larger number of degrees of freedom and closer to real life applications should be explored. The stability of cMTP decoders should be particularly evaluated. Transfer learning between BCI paradigms could be explored for the cMTP decoder training.

There is no technical limitation to using the pseudo-online implementation of the aaBCI framework performed in this study for actual online experiments. The online test of the proposed aaBCI approach in clinical trial experiments with tetraplegic subject is the nearest perspective of the study. The aaBCI used in this study is also compatible with at-home usage in the future, as it can run in real-time on a consumer-grade computer with a fast update rate (every 15 s).

The results of this proof of concept of aaBCI are encouraging for the development of BCIs usable not only in laboratories but also in patients’ daily environments over extended periods. The increasing number of chronic BCI clinical trials^2,18,30–32^ should make aaBCI studies easier to perform in the future and allow replicating and improving the results presented in this case study.

## Materials and methods

### Participant and signal acquisition

The participant in this study was a 28-year-old man who had tetraplegia following a spinal cord injury. The subject was implanted with two WIMAGINE^33^ ECoG implants in the scope of the clinical trial “BCI and Tetraplegia” (ClinicalTrials.gov identifier: NCT02550522, registered on the 15/09/2015) (Fig. 2A). The clinical trial was approved by French authorities: National Agency for the Safety of Medicines and Health Products (Agence nationale de sécurité du médicament et des produits de santé, ANSM) with the registration Number: 2015-A00650-49 and the ethical committee for the Protection of individuals (Comité de Protection des Personnes—CPP) with the Registration number: 15-CHUG-19. All research activities were carried out in accordance with the guidelines and regulations of the ANSM and the CCP, and the patient signed informed consent prior to surgery. Details of the clinical trial are available in Ref.^2^.

Two WIMAGINE recording systems were implanted epidurally into the skull within a 25 mm radius craniotomy and positioned over the right and left sensorimotor cortex (Fig. 2B)^2^. WIMAGINE implants are designed for chronic BCI applications and wireless data transmission^33^. The ECoG signals are registered from 64 electrodes, band pass filtered in a bandwidth 0.5–300 Hz and radiotransmitted to a custom designed base station at 586 Hz. Data from 32 electrodes per implant are transmitted due to limited data transfer rates. The 32 electrodes were chosen in a checkerboard-like pattern to maximize the span of the recording area over the sensorimotor cortex.

### Experiments

The participant took part in two series of experiments for this study. In each experimental session, the participant used a motor-imagery-based BCI to control a virtual effector displayed on a computer screen.

In a first series of experiments (paradigm 1), the participant controlled the movements of a virtual exoskeleton. This virtual exoskeleton was displayed on the computer screen from the point of view of the human avatar in the exoskeleton (Fig. 2C). The avatar could be either idle or in one of four mutually exclusive movement states: movement of the right or left hand or rotation of the right or left wrist. The subject activated and maintained each motor state with motor imagery. Visual information was presented on the screen to let the participant know which state he should activate and maintain. Ten sessions of approximately 20 min were acquired over the course of three months (starting 28 months after implantation).

In a second series of experiments (paradigm 2), the participant controlled a hand-shaped cursor on the computer screen in a two-dimensional center-out task. The cursor could move continuously in two dimensions and the participant controlled both its direction and its speed, up to a saturation value of 0.08 m s^−1^. The direction and speed of the cursor were updated every 0.1 s. In each center-out trial, the cursor had to reach one of eight possible targets (Fig. 2D). Nineteen sessions of approximate average length 16 min were acquired over the course of one and a half month (starting two and a half months after implantation).

### Neural feature extraction

Time–frequency information is known to be suited to motor imagery tasks with most of the information being in the frequency band below 200 Hz^34^. On the other hand, little is known regarding neural feature extraction for a continuous in time signal correlated to the perceived correctness of actions performed by a BCI-controlled effector. The use of time–frequency information for the cMTP decoder stems from several studies in which event-locked error correlates were detected in the motor cortex using time frequency information^20,22,23^. Epochs of one second were considered for feature extraction for both the cMTP decoder and the motor control decoders. These epochs were spaced by 0.1 s (90% overlap). For each epoch $t$, time–frequency information was extracted for each electrode using continuous complex wavelet transform (Morlet) with central frequencies ten hertz apart from each other, from 10 to 150 Hz. Absolute values were averaged in ten non-overlapping windows of 100 ms. Resulting feature tensors were noted ${\underline{\varvec{X}}}^{t}\in {\mathbb{R}}^{\tau \times f\times s}$, with $\tau =10$, $f=15$ the number of analyzed frequencies and $s=64$ the number of recording electrodes. The data tensor of all samples is noted $\underline{\varvec{X}}\in {\mathbb{R}}^{N\times \tau \times f\times s}$, with $N$ the total number of epochs for a given series of experiments.

### Training datasets for the supervised training of the control decoders

In this study, control decoders were trained supervisedly at two occasions. Once during classical online training sessions to be able to acquire the datasets (the data from these training sessions was not reused in the analysis), and once during the simulation of online use of the aaBCI for comparison purposes. Training data set is composed by tensor of neural features $\underline{\varvec{X}}\in {\mathbb{R}}^{N\times \tau \times f\times s}$, and corresponding labels. For the control decoders of the multi-class discrete BCI (paradigm 1), the output label ${{{\varvec{y}}}^{t}}_{control}$ for each epoch $t$ was a vector containing a dummy encoding of the class label, the desired state at the end time moment of the epoch. For the continuous control decoder (paradigm 2), the output label ${{{\varvec{y}}}^{t}}_{control}$ for each epoch $t$ contained Cartesian directed distance from the cursor to the target. We noted the matrix of control decoder real labels as ${{\varvec{Y}}}_{control}=($ ${{{\varvec{y}}}^{1}}_{control}, \dots , {{{\varvec{y}}}^{N}}_{control}{)}^{T}\in {\mathbb{R}}^{N\times m}$. Here, $m=4$ (the number of classes) for paradigm 1, and $m=2$ (the number of degrees of freedom) for paradigm 2; $N$ is the total number of epochs.

### Training datasets for cMTP decoders

cMTP decoders were trained supervisedly. Similarly, training datasets were composed by a tensor of neural features and corresponding labels. The output label ${{y}^{t}}_{cMTP}$ for each epoch $t$ was a binary value regarding the correctness of the BCI feedback. The output label reflects the coherence of actions performed by the BCI and the user’s intention/instruction. For the multi-class discrete BCI (paradigm 1) these labels were obtained by comparing the state of the virtual exoskeleton to the desired one. Epochs were labeled as errors with ${{y}^{t}}_{cMTP}=0$ (referred as ‘error class’) if the exoskeleton movement state was different from the desired one and correct with ${{y}^{t}}_{cMTP}=1$ (referred as ‘correct class’) if the states were similar. For the continuous control BCI (paradigm 2), epochs were labeled as correct or erroneous depending on the angle between the displacement performed at the previous time step by the cursor and the optimal displacement that could have been performed directly toward the target. This angular deviation was averaged over a sliding window of 0.5 s. When the mean angular deviation was higher than 90° the epoch was labeled as an error. When the mean angular deviation was lower than 60° the epoch was labeled as correct. These angles were chosen based on discussions with the subject regarding which movements they considered correct or erroneous. We noted ${{\varvec{y}}}_{cMTP}={( {{y}^{1}}_{cMTP},\dots , {{y}^{N}}_{cMTP})}^{T}\in {\mathbb{R}}^{N}$ the vector of all real cMTP decoder labels.

Supplementary inclusion/exclusion rules were applied to ensure that cMTP decoders were trained using the most relevant data. Epochs for which the real cMTP was not certain were removed. For paradigm 2, this is achieved by removing epochs for which the mean angular deviation was between 60° and 90°. For paradigm 1, the first 500 ms after a change of state of the exoskeleton was considered as uncertain due to reaction time. Indeed, the user needs some time to see that the state of the avatar changed and then internalize the change in cMTP. Considering classical motor reaction times and the temporality of known discrete motor task performance neural correlates, we estimated that the cMTP uncertainty would last less than 500 ms. To ensure that there was no data leakage from one class to another, epochs that contained neural data from 0 to 500 ms after a change in the state of a discrete effector were not included in the training set of the cMTP decoders.

**Figure 4:**
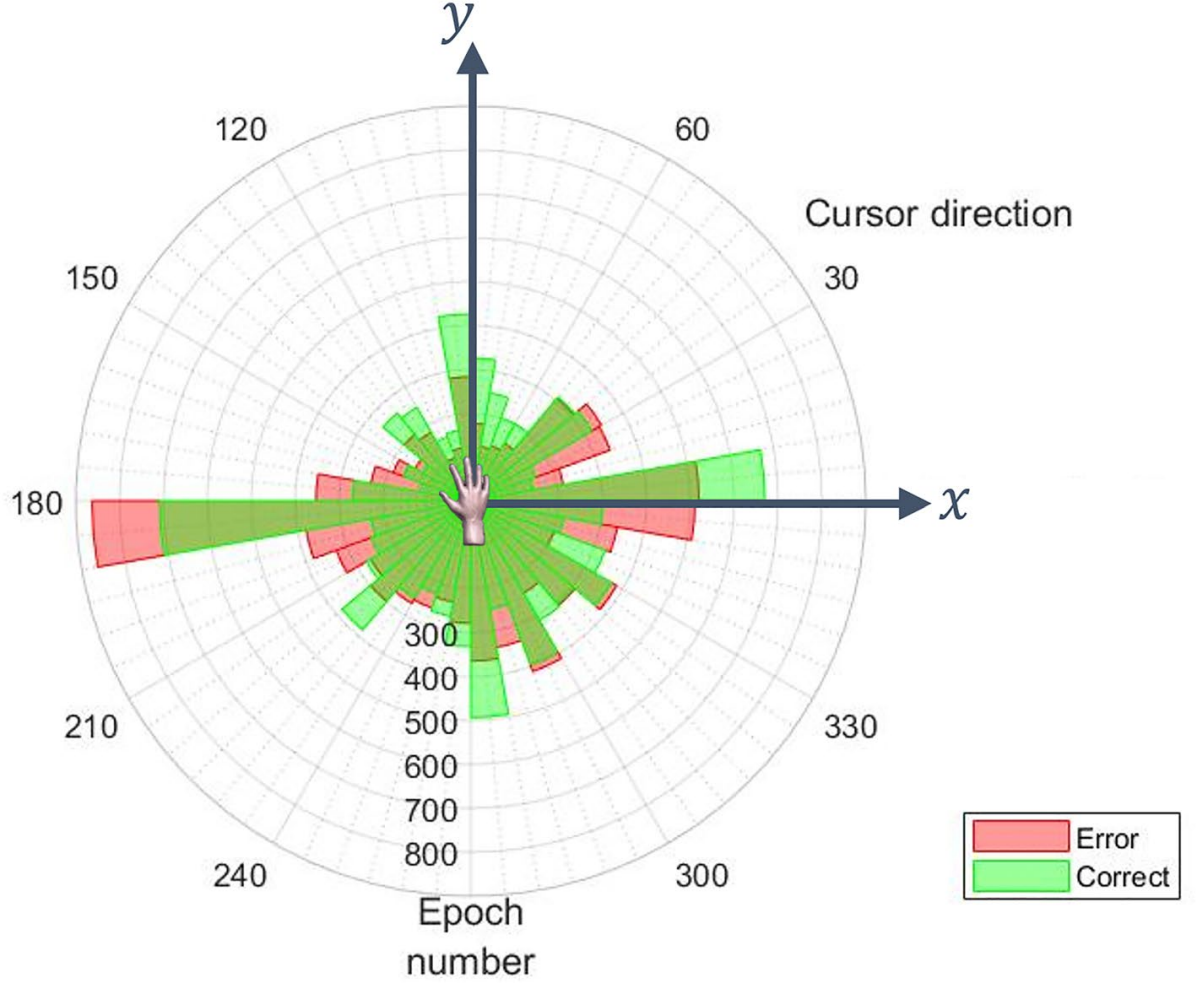
Distribution of direction of cursor displacement for the correct and error classes in the training dataset of the cMTP decoder (for one fold).

An additional concern regarding the creation of training datasets for cMTP decoders is the possibility of confounds with motor tasks. In this study, the user performed motor imagery to control the effectors. To be as close as possible to free use of BCI the number or type of motor errors that occurred while the user controlled the BCI was not influenced. This resulted in distributions of performed motor actions that could vary between the two cMTP classes. A solution we used to prevent motor confounds was to balance the motor actions found in each cMTP class to reach a similar uniform distribution. For the multi-class discrete paradigm, oversampling was performed by repetitions of samples that were in infrequent motor classes. For the continuous BCI paradigm, motor actions could not be well separated due to their continuous nature and no oversampling was performed. However, the cursor movement distributions were fairly similar between the correct and erroneous classes (Fig. 4).

### Formation of datasets for the training of control decoders in the auto-adaptive BCI framework

In the auto-adaptive framework, the output of the cMTP decoder is used to label data and create training datasets to update regularly the control decoder. As the cMTP decoder is not expected to have perfect accuracy, the control labels obtained using the cMTP may differ from the actual labels ${{\varvec{Y}}}_{control}$. These estimated labels are noted ${\widetilde{{\varvec{Y}}}}_{control}=($ ${{\widetilde{{\varvec{y}}}}^{1}}_{control}, \dots , {{\widetilde{{\varvec{y}}}}^{N}}_{control}{)}^{T}\in {\mathbb{R}}^{N\times m}$.

In order to maximize the effectiveness of the aaBCI decoder training, only samples that could be classified as erroneous or correct with enough certainty were included in the newly formed training datasets. To do so, the output of the cMTP decoder $${\widehat{{\varvec{y}}}}_{cMTP}$$ on the training set was modeled as a mixture of two Gaussian distributions, $$\mathcal{N}({\mu }_{err},{{\sigma }_{err}}^{2})$$ for the error class ($${{y}^{t}}_{cMTP}=0$$) and $$\mathcal{N}({\mu }_{corr},{{\sigma }_{corr}}^{2})$$ for the correct class ($${{y}^{t}}_{cMTP}=1)$$. The parameters of the two Gaussians were estimated for each class. We defined the inclusion thresholds $${th}_{corr}$$ and $${th}_{err}$$ as $${th}_{corr}={\mu }_{corr}+ {a \sigma }_{corr}$$ and $${th}_{err}={\mu }_{err}- {a \sigma }_{err}$$. Samples for which $${{\widehat{y}}^{t}}_{cMTP}>{th}_{corr}$$ were considered correct. Samples for which $${{\widehat{y}}^{t}}_{cMTP}< {th}_{err}$$ were considered erroneous. Samples for which $${th}_{err}<{{\widehat{y}}^{t}}_{cMTP}<{th}_{corr}$$ were not included into the new training sets. The constant $$a$$ balanced the trade-off between the number of samples added to the training set and the confidence of their labelling by the cMTP decoder. Although the hyperparameter $$a$$ could be optimized, we set $$a=1$$ in this proof-of-concept study.

For an update $u$ the estimated control labels ${{\widetilde{{\varvec{Y}}}}^{u}}_{control}$ were obtained from a relabeling function $\varphi$ using the output of the cMTP decoder ${{{\widehat{{\varvec{y}}}}^{u}}_{cMTP}=( {{\widehat{y}}^{{t}_{1}}}_{cMTP},\dots , {{\widehat{y}}^{{t}_{n}}}_{cMTP})}^{T}$ and the output of the control decoder ${{\widehat{{\varvec{Y}}}}^{u}}_{control}=({{\widehat{{\varvec{y}}}}^{{t}_{1}}}_{control}, \dots , {{\widehat{{\varvec{y}}}}^{{t}_{n}}}_{control}{)}^{T}$, with $({t}_{1},\dots , {t}_{n})$ the time points between update $u-1$ and update $u$:

$${{\widetilde{{\varvec{y}}}}^{t}}_{control}= \varphi \left({{\widehat{y}}^{t}}_{cMTP}, {{\widehat{{\varvec{y}}}}^{t}}_{control}\right)$$

We used two re-labelling functions ${\varphi }_{1}$ and ${\varphi }_{2}$ depending on the BCI paradigm. For paradigm 1, ${\varphi }_{1}:\left]-\infty ;{th}_{err} \right]\cup \left[{th}_{corr};\infty \right[\times {\mathbb{R}}^{m} \mapsto {\mathbb{R}}^{m}$ was defined as:

$${\varphi }_{1}\left({{\widehat{y}}^{t}}_{cMTP}, {{\widehat{{\varvec{y}}}}^{t}}_{control}\right)=\left\{\begin{array}{c}\begin{array}{l}{{\varvec{e}}}_{{c}_{1}^{t}}\\ {{\varvec{e}}}_{{c}_{2}^{t}}\end{array}\end{array}\right. \begin{array}{c}, if {{\widehat{y}}^{t}}_{cMTP}>{th}_{corr} \\ , if {{\widehat{y}}^{t}}_{cMTP}< {th}_{err}.\end{array}$$

Here $m$ is the number of classes, ${{({\varvec{e}}}_{i})}_{i=1,\dots ,m}$ is the canonical base of ${\mathbb{R}}^{m}$ and ${c}_{k}^{t}$ the *k*th most probable class estimated by the control decoder for sample $t$, defined as:

$$k \in \left\{ {2, \ldots ,m} \right\},\begin{array}{*{20}l} {c_{1}^{t} = \mathop {\arg \max }\limits_{{i \in \{ 1, \ldots ,m\} }} \hat{y}_{{control}}^{t} ,} \hfill \\ {c_{k}^{t} = \mathop {\arg \max }\limits_{{i \in \{ 1, \ldots ,m\} \backslash \{ c_{1}^{t} , \ldots ,c_{{k - 1}}^{t} \} }} \hat{y}_{{control}}^{t} .} \hfill \\ \end{array}$$

The output of the relabeling function ${\varphi }_{1}$ corresponds to the dummy encoding of the most probable class (if the sample was estimated correct) or the second most probable class (if the sample was estimated erroneous).

For the continuous BCI (paradigm2) ${\varphi }_{2}:\left[{th}_{corr};\infty \right[\times {\mathbb{R}}^{m} \mapsto {\mathbb{R}}^{m}$ ($m$ being the number of continuous outputs of the control decoder) was defined as:

$${\varphi }_{2}\left({{\widehat{y}}^{t}}_{cMTP}, {{\widehat{{\varvec{y}}}}^{t}}_{control}\right)={{\widehat{{\varvec{y}}}}^{t}}_{control}.$$

The relabeling function ${\varphi }_{2}$ only keeps samples that were estimated corrects, with the label being the one estimated by the control decoder.

### Algorithms for decoders training

The control decoders used to acquire the datasets were trained online using the Recursive Exponentially Weighted N-way Partial Least Squares algorithm (REW-NPLS)^25^, employed for closed loop decoder training in this clinical trial^2^.

The cMTP decoders of the aaBCI were trained offline using the N-way Partial Least Squares (NPLS), with a fixed number of factors of 20 based on preliminary studies.

The control decoders of the aaBCI were trained in a simulation of online use. The aaBCI requires an algorithm that can train BCI control decoders adaptively in real-time. Therefore, the adaptive REW-NPLS was used to train the control decoders taking advantage of the labels estimated from the cMTP decoders. Updates of the control decoders were performed each time 15 s of labeled data was acquired. The number of factors of the REW-NPLS was optimized after each update^25^. The REW-NPLS and the NPLS are particularly adapted to the tasks at hand, since they are well-suited to tensor-shaped, high dimensional datasets (9600 input features) and can be used for both classification and regression^24,25^. REW-NPLS is a supervised learning algorithm, however the update of the control decoder in the aaBCI framework can be considered as unsupervised as labels in training data are not delivered from outside.

For comparison purposes, the simulations of supervised control decoders training were carried out using the same REW-NPLS algorithm applied to the training data with true labels. Updates of the control decoders were performed each 15 s.

### aaBCI framework performance evaluation

Performance of the proposed aaBCI framework was evaluated for two different tasks for each BCI paradigm. The first task was the detection of cMTP neural correlates by the cMTP decoder. The second task was the training of the control decoder using the auto-adaptive BCI framework. The decoding capabilities of the cMTP decoder were assessed using the mean Area Under the Curve (AUC) of its Receiver Operating Characteristic (ROC) curve over the test folds of a fivefold cross-validation. The cross-validation was performed by session. Each fold contained data from the same number of recording sessions. To estimate the performances of the auto-adaptive BCI, each dataset was separated in three non-overlapping splits, each split containing the same number of recording sessions (up to one session difference). One split was used to train the cMTP decoder, one split was used to train the control decoder and one split was used to test the performances of the control decoder. In real use-cases, the data used to train the cMTP decoders could be used to pre-train the control decoder as well. In this proof-of-concept study, we decided to train the control decoders from scratch using only the aaBCI framework in order to emphasize its ability to train control decoders. Control decoders were thus initialized with zeros. Permuting the roles of each split led to six performance measures for each effector. The performance of the control decoder was assessed on test splits with a different performance criterion for each effector. The discrete multi-class effector’s performance was evaluated using a generalized version of the AUC of the ROC curve for multi-class classification^26^. The continuous effector’s performance was evaluated using the cosine similarity between the predicted trajectory and the optimal trajectory to reach the current target. The performance of the auto-adaptive BCI for each effector was assessed with the mean of its associated performance criterion over each test split.

### Significance statement

Most brain–computer interfaces (BCIs) require strong time investments before they can be used because they require labeled neural data in order to know and optimize their performances. This investment to obtain labeled data often has to be repeated to maintain good performances. We propose a solution to this issue by giving BCIs a way to quantify by themselves their performance levels using brain signals from the user, and then to adapt themselves autonomously using this knowledge. The methods used in this proof of concept can be used for various motor BCIs, but are especially valuable for BCIs that control complex motor actions since they have different constraints than classical BCIs and did not have any adaptive solution yet.

## Data Availability

The datasets analyzed during the current study are not publicly available due to privacy concerns but are available from the corresponding author on reasonable request.
